# Migratory and adhesive properties of *Xenopus laevis* primordial germ cells *in vitro*

**DOI:** 10.1242/bio.20135140

**Published:** 2013-11-06

**Authors:** Aliaksandr Dzementsei, David Schneider, Andreas Janshoff, Tomas Pieler

**Affiliations:** 1 Department of Developmental Biochemistry, Göttingen Center for Molecular Biosciences, Georg-August-University, Justus-von-Liebig-Weg 11, 37077 Göttingen, Germany; 2 Institute of Physical Chemistry, Georg-August-University, Tammannstrasse 6, 37077 Göttingen, Germany

**Keywords:** Primordial germ cells, Cell migration, Cell adhesion, Cellular dynamics, *Xenopus*

## Abstract

The directional migration of primordial germ cells (PGCs) to the site of gonad formation is an advantageous model system to study cell motility. The embryonic development of PGCs has been investigated in different animal species, including mice, zebrafish, *Xenopus* and *Drosophila*. In this study we focus on the physical properties of *Xenopus laevis* PGCs during their transition from the passive to the active migratory state. Pre-migratory PGCs from *Xenopus laevis* embryos at developmental stages 17–19 to be compared with migratory PGCs from stages 28–30 were isolated and characterized in respect to motility and adhesive properties. Using single-cell force spectroscopy, we observed a decline in adhesiveness of PGCs upon reaching the migratory state, as defined by decreased attachment to extracellular matrix components like fibronectin, and a reduced adhesion to somatic endodermal cells. Data obtained from qPCR analysis with isolated PGCs reveal that down-regulation of E-cadherin might contribute to this weakening of cell-cell adhesion. Interestingly, however, using an *in vitro* migration assay, we found that movement of *X. laevis* PGCs can also occur independently of specific interactions with their neighboring cells. The reduction of cellular adhesion during PGC development is accompanied by enhanced cellular motility, as reflected in increased formation of bleb-like protrusions and inferred from electric cell-substrate impedance sensing (ECIS) as well as time-lapse image analysis. Temporal alterations in cell shape, including contraction and expansion of the cellular body, reveal a higher degree of cellular dynamics for the migratory PGCs *in vitro*.

## Introduction

As germ cell specification takes place in a region different from the site of gonad formation, PGCs have to migrate in a directional manner during embryogenesis. In *Xenopus laevis*, germ cells are specified by inheriting maternal determinants that are part of the germ plasm. As a result of directional transport during oogenesis, this material localizes to the vegetal pole of the fertilized egg which contains a set of specific vegetally localizing mRNA known to be essentially involved in germ cell specification and migration (Berekelia et al., 2005). Followed by passive movement together with the surrounding endodermal cells during gastrulation, PGCs start to migrate actively within the endoderm at tailbud stages. Later, via a thin strip of connective tissue called dorsal mesentery, PGCs travel to the dorsal body wall where they associate with somatic gonadal cell precursors. Finally, the primordial germ cells form germ line stem cells, which differentiate into the gametes (Heasman et al., 1977; Molyneaux and Wylie, 2004; Wylie et al., 1976; Wylie and Heasman, 1976).

The transition of *X. laevis* PGCs to active migration in the endoderm occurs after stage 24 of the development (Nishiumi et al., 2005). In the context of this transition, cells start to disperse from a cluster that they had formed before. In comparison to the pre-migratory stages, most of the PGCs isolated after the transition to active migration exhibit an altered cell morphology. Their shape changes from spherical with small bleb-like protrusions to elongated or irregular with larger protrusions (Terayama et al., 2013).

It seems that cell-blebbing is the basis for migration of PGCs in zebrafish, *Drosophila* and also *Xenopus laevis* (Blaser et al., 2005; Jaglarz and Howard, 1995; Wylie, 1999). Blebs are spherical plasma membrane protrusions, whose formation is pressure-driven and not supported by the actin cytoskeleton. In non-motile cells, subsequent to bleb formation, a myosin II-driven retraction of the protrusion occurs mediated by cortical actin. In migrating cells, retraction of the bleb at the leading edge does not occur and an actin-myosinII dependent contraction of the cell body pushes the cytoplasm in the direction of a stabilized bleb at its leading edge (Charras et al., 2008). A similar mode of motility is observable for tumor cells during protease-independent migration (Wolf et al., 2003). It has remained unclear, how exactly bleb-formation is involved in the process of cellular migration. One theory describes a weak adhesion of migrating cells to the extracellular matrix and the surrounding cells, allowing cellular movement due to the contraction of the cell rear and the concomitant disassembly of primary cell-ECM or cell-cell interactions (Charras and Paluch, 2008). According to a second hypothesis, cells are capable of exerting forces perpendicular to the top and bottom substrate, thereby pushing themselves forward. This type of migration - called chimneying - is independent of cellular adhesion (Malawista et al., 2000). Migration of PGCs has been studied extensively in different vertebrate and invertebrate systems (reviewed by Richardson and Lehmann, 2010). More recent work in the zebrafish has uncovered that migrating PGCs form E-cadherin mediated contacts with neighboring somatic cells. These interactions are required to generate traction force for cell migration (Kardash et al., 2010). The level of E-cadherin expression, however, is down-regulated in comparison to pre-migratory PGCs in order to allow for a fast turnover of adhesion contacts (Blaser et al., 2005; Goudarzi et al., 2012; Kardash et al., 2010). Thus, changes in the migratory behavior of cells during embryogenesis may not be solely due to increased formation of cellular protrusions, but they could also be caused by differences in the expression or stability of adhesion molecules.

A pioneering study by Heasman and coworkers described the functional relevance for fibronectin on PGC migration in dorsal mesentery in *Xenopus* embryos (Heasman et al., 1981). In addition, β1-integrins are also thought to contribute to active migration of PGCs (Nishiumi et al., 2005). However, questions regarding the overall strength of cellular adhesion, the occurring changes on the molecular level upon reaching the migratory stage, as well as a quantification of cellular motility are so far only fragmentarily addressed.

In the context of this study, we investigated the adhesive and dynamic behavior of *Xenopus laevis* PGCs before and after their transition to the active migratory state. Using single-cell force spectroscopy (Helenius et al., 2008), we reveal a stronger interaction of pre-migratory PGCs (stage 17–19 of *X. laevis* development) with the extracellular matrix components fibronectin as compared to migratory PGCs (stage 28–30). The apparent decline of attachment to the extracellular matrix during development is accompanied by a decrease in adhesiveness to the surrounding somatic cells. This change in adhesive properties correlates with a down-regulation of E-cadherin mRNA level, in migratory PGCs. Furthermore, via electric cell-substrate impedance sensing (Giaever and Keese, 1984) and concomitant time-lapse image analysis, we could show that cellular dynamics of PGCs increase during embryogenesis. In summary, we propose that reduced cell adhesion and a concomitant increase in cellular dynamics is of relevance for the initiation of active migration of PGCs during embryonic development in *Xenopus laevis*.

## Results

### Alterations in adhesive behavior of primordial germ cells and somatic endodermal cells during early embryonic development

**Fig. 1.**
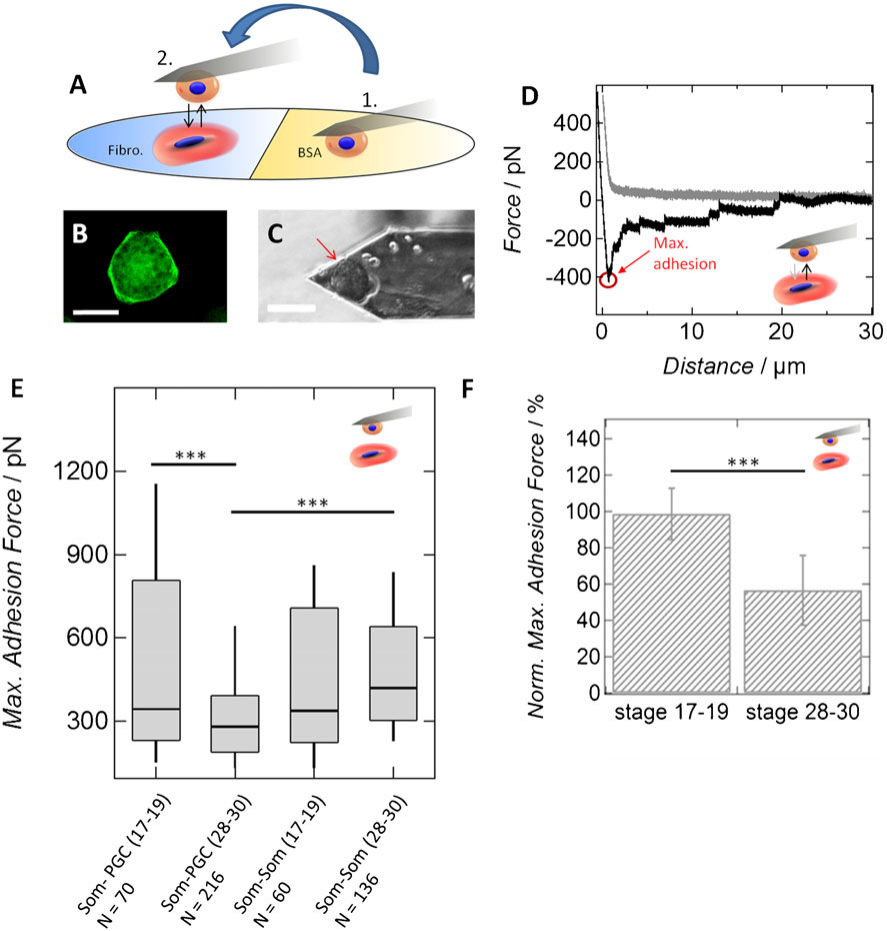
PGCs reduce overall cell-cell adhesion after transition to the active migration state. (A) Cells were isolated from *GFP_DELE* mRNA injected embryos and transferred to the Petri dish half coated with fibronectin and half coated with bovine serum albumin (BSA). Weakly adhering cells from BSA-coated region were attached to an atomic force microscope cantilever (1). Subsequently, the attached cell is brought into contact with a cell spread on fibronectin-coated part of the Petri dish (2). (B) Fluorescence image of a labeled primordial germ cell spread on a fibronectin-coated Petri dish. (C) Bright-field image of a migratory PGC attached to a poly-D-lysin coated cantilever. Scale bars: 50 µm. (D) Example of a force-distance curve of two somatic cells from the same developmental stage. The approach curve (grey), as well as the retraction curve (black) are shown. (E) Maximum adhesion force either between PGCs and somatic endodermal cells (PGC-Som) or between two somatic endodermal cells (Som-Som) isolated from embryos at stages 17–19 (pre-migratory PGCs) or stages 28–30 (migratory PGCs). Box-whisker plots: lines reflect the median of the distribution, boxes comprise the 25th and 75th percentile, whisker tops and bottoms are drawn to the 10th and 90th percentiles, respectively. *N* corresponds to the number of curves that have been analyzed per category. *** corresponds to *P*-values<0.001 (Wilcoxon rank sum test). The number in brackets describes the stage of PGC development: 17–19: pre-migratory PGCs and 28–30: migratory PGCs. (F) Mean maximum adhesion force of either pre-migratory (stage 17–19, *n* = 4) or migratory (stage 28–30, *n* = 7) PGCs after contact with a somatic cell from the corresponding stage. Here *N* corresponds to the number of cells per category. For each cell at least 4 force curves have been recorded. For each PGC-somatic cell interaction, values are normalized to the interaction between the same somatic cell and a second somatic cell. *** corresponds to *P*-value<0.001 (Wilcoxon rank sum test). All p-values are given in supplementary material Table S1.

As regulation of cellular adhesion is known to play an important role in the context of germ cell migration in different animal species (Richardson and Lehmann, 2010), we set out to characterize the situation for PGCs in *Xenopus laevis*. For this purpose, we performed single-cell force spectroscopy (SCFC) studies of PGCs and somatic endodermal cells from pre-migratory and migratory stages of development (Fig. 1A). SCFS measures adhesive forces between two cells in contact or between a single cell and an artificial substrate, respectively. Active migration of *X. laevis* germ cells in the endoderm starts approximately at developmental stage 24 (Blaser et al., 2005). Hence, we used cells isolated from embryos at developmental stage 17–19 (neurula) for pre-migratory PGCs and cells isolated from embryos at stage 28–30 (tailbud) representing the migratory state. *Xenopus laevis* PGCs can be specifically labeled by the injection of mRNA containing GFP ORF fused to the *Xenopus Dead End* (*dnd1*) Localization Element (DELE) (Horvay et al., 2006; Koebernick et al., 2010). Therefore, cells were isolated from *GFP_DELE* mRNA injected embryos to distinguish between GFP-negative somatic endodermal cells and GFP-positive PGCs (Fig. 1B). Fig. 1C shows a single primordial germ cell isolated from a neurula stage embryo; the cell is attached to a poly-D-lysin coated tip-less cantilever as described in the methods section. By using single-cell force spectroscopy, we were able to quantify the interaction of PGCs with either somatic endodermal cells or with extracellular matrix components, respectively. As a measure of dynamic strength, force-distance curves were recorded and the maximum adhesion force upon retraction of the cantilever was determined (Fig. 1D). We found a significant decrease in the interaction forces between migratory PGCs and somatic cells, as compared to the interactions of PGCs and somatic cells earlier in development (Fig. 1E; supplementary material Table S1). In order to avoid effects that would be due to differences between individual cells, the maximum adhesion force recorded during interaction of a PGC with a somatic cell was normalized to the maximum adhesion force obtained during attachment of the same somatic cell with a second somatic one. Using this approach, the effect becomes even more obvious, although instability of the cells on the cantilever over long periods of time makes it difficult to perform a larger number of independent experiments (Fig. 1F). Taken together, these observations reveal that PGCs reduce their adhesiveness to the surrounding somatic cells upon transition to migratory stage. Interestingly, the adhesive strength between pre-migratory PGCs and somatic cells is similar to that of somatic cells with each other. In addition, the maximum adhesion force between two somatic cells from either the early or the late stage showed no significant differences in absolute values. Therefore, it is conceivable that, within the endodermal cell mass, alterations concerning adhesive properties are restricted to primordial germ cells.

**Fig. 2.**
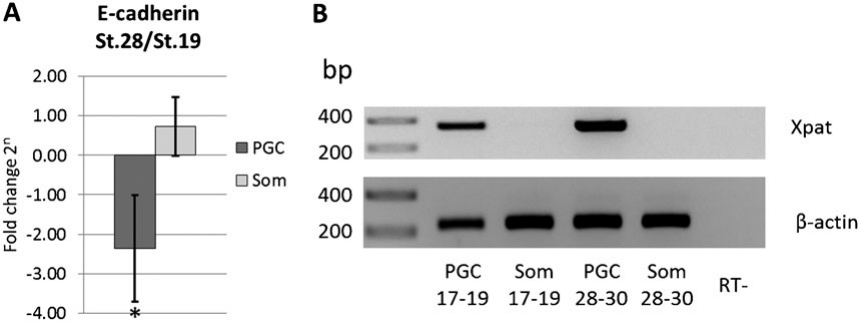
E-cadherin is downregulated in migratory PGCs. (A) Relative amount of E-cadherin (*cdh1*) in PGCs and somatic endodermal cells (Som) isolated from stage 28–30 embryos (migratory PGCs) normalized to E-cadherin level in the corresponding cell type isolated from stage 17–19 embryos (pre-migratory PGCs) measured by quantitative RT PCR. Relative amount was calculated by ΔΔCt method (see Material and Methods) using three independent cDNA preparations. Error bars represent standard deviation. * corresponds to *P*<0.05 (two-tailed t-test). (B) Agarose gel electrophoresis of PCR quality control for cDNA used in qPCR analysis. In contrast to somatic cells, PGC-specific cDNA contains *Xpat* transcript. Amplification of β-actin is used as a positive control; a sample obtained from reverse transcription of endodermal cells without adding reverse transcriptase (RT-) was used as a negative control. Marker lane on the left side of the gel indicates the relative size of amplified products in base pairs (bp).

One of the candidate adhesion molecules, which might be involved in the changes of cell-cell adhesion properties of PGCs, is E-cadherin. It was shown to be down-regulated during the onset of PGC migration in zebrafish (Blaser et al., 2005; Kardash et al., 2010; Goudarzi et al., 2012) and also to be involved in PGC development in mouse and *Drosophila* (De Felici et al., 2005; Kunwar et al., 2008; Richardson and Lehmann, 2010). We quantified the expression levels of E-cadherin by qPCR analysis of *X. laevis* PGCs and somatic endodermal cells isolated from neurula and tailbud stages. After the transition to active migration, E-cadherin was found to be down-regulated in PGCs, as opposed to somatic cells (Fig. 2).

**Fig. 3.**
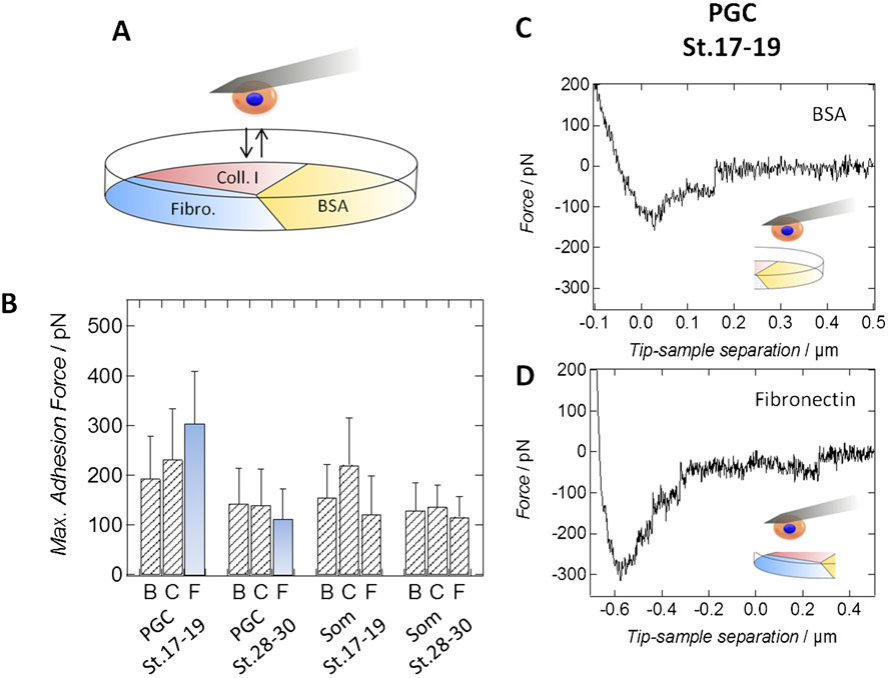
Pre-migratory PGCs show high affinity to fibronectin. (A) Schematic drawing of a Petri dish being divided into three sectors coated either with fibronectin, collagen I or bovine serum albumin (BSA). Either PGCs or somatic cells from both stages were brought into contact with the different substrates using single-cell force spectroscopy. (B) Maximum adhesion forces of either PGCs or somatic cells (Som) isolated from *GFP_DELE* mRNA injected embryos at developmental stage 17–19 (pre-migratory PGCs) or stage 28–30 (migratory PGCs) during interaction with BSA (B), collagen I (C) or fibronectin (F) coated surfaces. The most relevant change in interaction, the decline in binding strength of PGCs to fibronectin during development, is shown in blue bars. At least 37 force curves per category have been recorded and analyzed. Values are obtained from AFM force-distance curves. Negative forces correspond to positive adhesion forces. Error bars show standard deviations. All p-values obtained from either Wilcoxon rank sum tests or two-tailed t-tests are given in supplementary material Table S2. (C,D) Typical AFM force-distance curves monitoring the interaction of a pre-migratory primordial germ cell with a (C) BSA-coated and a (D) fibronectin-coated part of the Petri dish.

The influence of the extracellular matrix on PGC migration in *X. laevis* has been described for the migration of these cells in the dorsal mesentery (García-Castro et al., 1997; Heasman et al., 1981). Previous studies also showed that PGCs at tailbud stages can interact with fibronectin (Tarbashevich et al., 2011). We decided to perform a quantitative study concerning the strength of interaction between PGCs and the extracellular matrix during the transition of these cells to active migration. Hence, the attachment of migratory PGCs from *Xenopus laevis* to collagen I and fibronectin was investigated using single-cell force spectroscopy. Additionally, application of bovine serum albumin (BSA) was used as a negative control, resulting in a saturation of nonspecific binding sites on the surface of the culture dish (Fig. 3A). From the AFM retraction curves (Fig. 3C,D), we infer a higher adhesiveness of early PGCs to fibronectin as compared to collagen I and BSA after a contact time of 5 sec, which is evident from higher values of the maximum adhesion force (Fig. 3B, *P*-values<0.01, two-tailed t-test). 6 h after cell seeding, pre-migratory PGCs started to adhere to the fibronectin-coated part of the Petri dish, whereas on collagen I and BSA, the cells remained only loosely attached. However, a slight increase in adhesiveness between PGCs isolated from neurula stage embryos and collagen I compared to their interaction with BSA was detected (*P*-value<0.05, two-tailed t-test). Similarly, early somatic cells show an increased binding strength to collagen I (*P*-value<0.01, Wilcoxon rank sum test). Furthermore, in case of PGCs from early stages of development, values of the maximum adhesion force are generally higher than those for other cell types. This finding is independent of the investigated substrate, implying per se a higher adhesiveness of primordial germ cells before their transition to the state of active migration. Pre-migratory PGCs show a nearly 3-fold stronger attachment to fibronectin than PGCs from the tailbud stage or somatic cells, as well as an almost 1.5-fold and 1.4-fold higher maximum adhesion force for collagen I and BSA, respectively (Fig. 3B, *P*-values<0.01, Wilcoxon rank sum test). Assuming that the interaction between PGCs and the bovine serum albumin coated surface serves as a useful negative reference, we can assign that the strength of interaction to the fibronectin-coated part of the Petri dish is about 2-fold higher for pre-migratory PGCs than for migratory PGCs. Pre-migratory PGCs also show higher specific binding to collagen I than migratory ones, a finding similar to the one made for early and late somatic cells (Fig. 3B, *P*-values<0.01, Wilcoxon rank sum test). We conclude that the overall adhesiveness of pre-migratory PGCs is higher compared to PGCs in the active migratory state (supplementary material Table S2), particularly regarding their interaction with the extracellular matrix protein fibronectin.

An increase in contact time to 30 sec leads to higher maximum adhesion forces as exemplarily inferred from force measurements with PGCs and somatic cells, both from the early developmental stage, adhering either to fibronectin or BSA (data not shown). However, the relative changes in adhesion during cellular development remain the same. Only PGCs show an increased adhesiveness to fibronectin in comparison to BSA opposed to somatic cells. Furthermore, the variance of the values rises with contact time, especially for early PGCs on fibronectin. This finding is attributed to the migratory behavior of the cells on their preferred substrate leading to a decline in significance of the obtained maximum adhesion forces in case of longer contact times. Therefore, we decided to analyze only force distance curves recorded with a contact time of 5 sec.

### Formation of specific cell-cell contacts and extracellular matrix adhesion are not required for bleb-associated migration of *X. laevis* PGCs *in vitro*

**Fig. 4.**
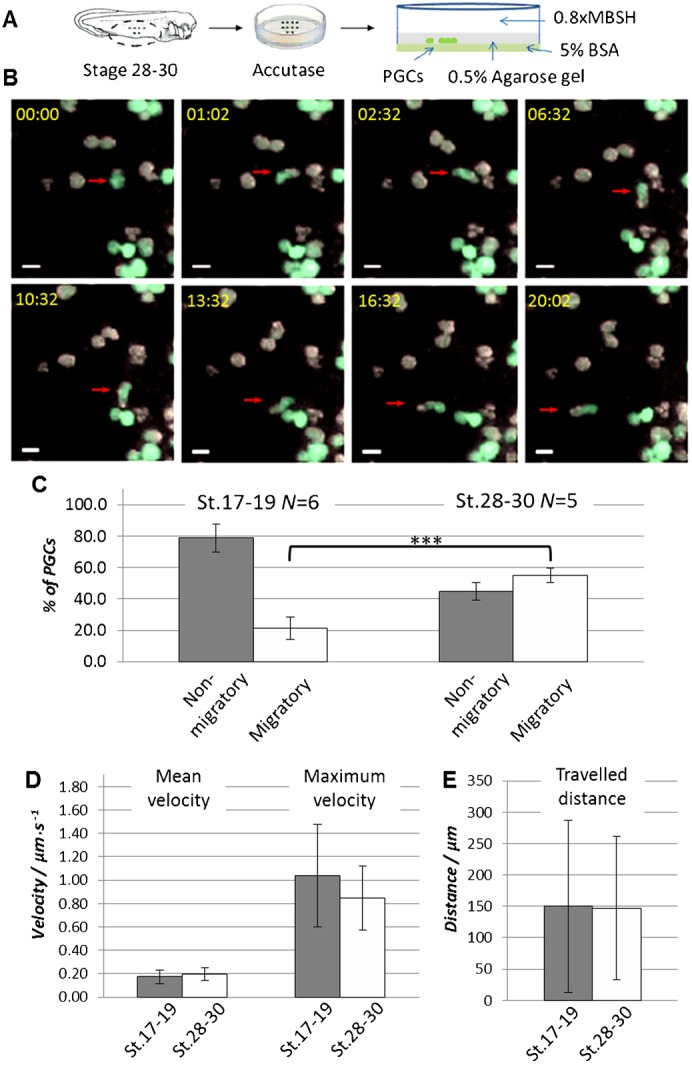
*X. laevis* PGC can migrate *in vitro* in the under-agarose migration assay. (A) Ventral explants were dissected from the embryos at developmental stage 17–19 or 28–30 injected at 2-cell stage vegetally with *GFP_DELE* mRNA to label PGCs. Explants were treated with accutase and the dissociated cells were transferred between 0.5% agarose gel and a 5% BSA-coated Petri dish in 0.8× MBSH buffer. (B) Time-lapse images of an under-agarose migration assay with dissociated endodermal cells from stage 28–30 embryos. PGCs can be identified as GFP-positive (green) in contrast to the GFP-negative somatic cells. Red arrows indicate migrating PGC. Relative time from the start of the time-lapse imaging is shown in the upper left corner of each image panel (min:sec). Scale bars: 20 µm. (C) Time-lapse microscopy was used to monitor the behavior of PGCs. Relative amount of migratory and non-migratory cells in the total amount of analyzed PGCs was calculated for each experiment. *N* – number of experiments. Error bars represent standard deviation. *** corresponds to *P*<0.001 (two-tailed t-test). (D,E) Migrating cells from stage 17–19 (*n* = 17) and stage 28–30 (*n* = 28) were used for the tracking analysis. Diagrams represent mean and maximum velocity of the cells (D) and distance which migrating PGCs travelled from the start (E). Error bars represent standard deviation.

The lack of transparency due to a high amount of the yolk granules in *X. laevis* endodermal cells makes it difficult to apply standard microscopy techniques to the *in vivo* analysis of PGC migration during tailbud stages of development. Therefore, we decided to develop an *in vitro* system to study the cellular mechanisms underlying this phenomenon. *X. laevis* PGCs migrate within the endoderm via a blebbing-associated mechanism (Tarbashevich et al., 2011). There are two major models describing how plasma membrane bleb-like protrusions can contribute to directional migration. One implies formation of focal adhesions between a newly formed bleb and the substrate. According to the second model, adhesion between the cell and the substrate is not required as long as the cell is surrounded by at least two resistant surfaces, which allow expansion of the bleb into one direction only (Charras and Paluch, 2008). Reproducible PGC migration was achieved using a so called “under-agarose” migration assay, very much in line with the assumptions of the second model. In these experiments, ventral explants containing labeled PGCs were isolated from the embryos at developmental stages 28–30. Explants were dissociated with accutase (a mild protease) and cells were transferred onto a BSA-coated Petri dish underneath a 0.5% agarose gel (Fig. 4A). BSA coating was used to prevent nonspecific binding of the cells to the plastic surface of the Petri dish, while an agarose gel cover served to create a second resistant surface. Migration of PGCs in this assay suggests that they do not require specific cell-cell or extracellular matrix adhesion for motility (Fig. 4B). Moreover, when migrating PGCs get into contact with another cell, new interactions form that anchor PGCs and prevent them from moving despite persisting cellular dynamics to resume or start migration (supplementary material Fig. S1).

We also used an under-agarose migration assay described above to test migratory abilities of PGCs isolated from neurula stage embryos (developmental stage 17–19) and to compare those to PGCs isolated from tailbud stage embryos (developmental stage 28–30). Surprisingly, some of the PGCs from the early developmental stage also showed migratory behavior (Fig. 4C; supplementary material Fig. S4). In case of PGCs isolated from tailbud stage embryos, however, the amount of migratory cells was increased to approximately 50%. Analysis of the migrating PGCs isolated from neurula and tailbud stage embryos showed no difference in mean and maximum velocity (Fig. 4D) or traveled distance (Fig. 4E). This suggests that, already at the neurula stage, PGCs can perform active migration, but they do so more avidly at the tailbud stage.

### Cellular dynamics of primordial germ cells

**Fig. 5.**
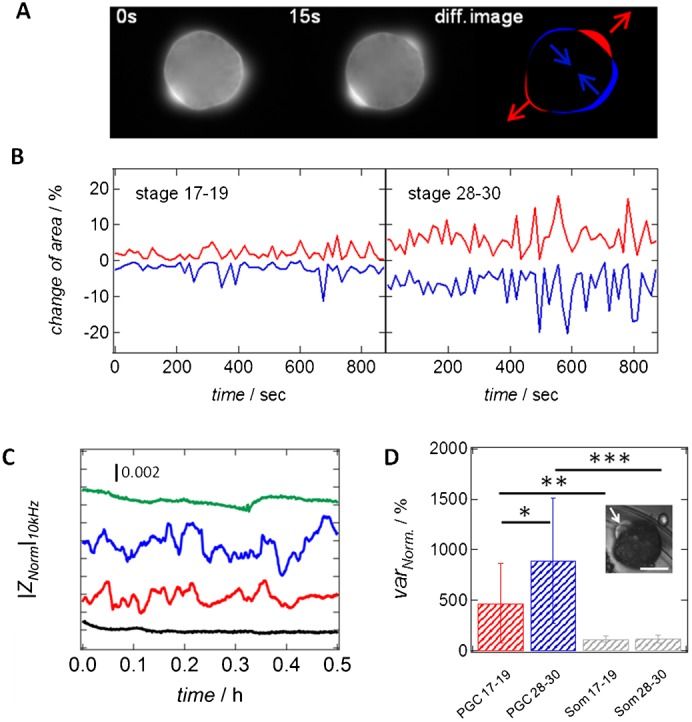
PGCs isolated from tailbud stage embryos show high cellular motility. (A) Time-lapse fluorescence images of GFP-labeled PGCs from the migratory stage were taken with a time interval of 15 s. Subtracting two successive images from each other results in a difference image showing the expanding (red) and contracting (blue) regions of the cell over time. Arrows underscore the direction of expansion/contraction. (B) Time traces of the contracting (blue) and expanding (red) area of PGCs from the pre-migratory (stage 17–19) and the migratory (stage 28–30). Values are normalized to the current cell area at each time point and given in percentages. (C) Time-resolved, normalized and detrended impedance data of an uncovered electrode (*black line*), a single pre-migratory primordial germ cell (PGC stage 17–19, *red line*), a single migratory primordial germ cell (PGC stage 28–30, *blue line*) and a migratory primordial germ cell treated with 100 µM cytochalasin B (PGC stage 28–30, *green line*). All measurements were recorded at an excitation frequency of 10 kHz. The curves are arbitrarily shifted in the y-direction for better visibility. (D) Calculated variances obtained from time-resolved impedance data (C) for single pre-migratory primordial germ cell (PGC stage 17–19, *n* = 7, *red*) and single migratory primordial germ cell (PGC stage 28–30, *n* = 6, *blue*). Additionally, normalized variances of somatic endodermal cells from both stages of embryonic development (stage 17–19 with *n* = 4 and stage 28–30 with *n* = 6) are shown in grey. Values are given in percentages and normalized to the variance of the time-trace of an uncovered electrode (100%). *N* depicts the number of investigated cells per category. Error bars represent standard deviation. * corresponds to *P*-value<0.05 (*P*-value = 0.079), ** corresponds to *P*-value<0.1 (*P*-value = 0.028), *** corresponds to *P*-value<0.01 (*P*-value = 0.001). In all cases a Wilcoxon rank sum test was performed. Inlay: Single migratory PGC (stage 28–30) showing a strong formation of blebs (indicated by white arrow). Scale bar: 10 µm.

To quantify the formation of bleb-like protrusions during PGC migration, we investigated cellular dynamics of PGCs before and after transition to the active migratory state using electric cell-substrate impedance sensing (ECIS), in conjunction with time-lapse image analysis. Image analysis, as described in the Materials and Methods section, reveals strong dynamics of PGCs, as reflected in the formation of bleb-like protrusions and significant shape fluctuations at both stages (Fig. 5A; supplementary material Figs S2, S3). PGCs from the migratory stage show a significantly higher tendency for expansion and contraction of the cellular body than pre-migratory PGCs (Fig. 5B). In order to quantify cellular dynamics, we calculated variances of the temporal shape fluctuations as shown in Fig. 5B and detected a 2.4-fold increase of the calculated variances upon onset of migration. Interestingly, areas of expansion are in most cases neighbored by contracting (Fig. 5A). Along the same lines, changes in the fluctuation amplitude of the detected impedance signal (Fig. 5C) reflect the movement of the cell on the electrode including bleb formation and retraction (Fig. 5D). This movement is commonly referred to as *micromotion*. Upon addition of cytochalasin B, an actin-depolymerizing agent and consequently an inhibitor of cellular motility, a strong decline in the impedance noise is detectable, finally reaching values comparable to those of an uncovered electrode. Furthermore, the amplitude of fluctuation increases in case of migratory PGCs, which is reflected in variance values being about 1.9-fold higher than those for PGCs from the early stage. Besides changes in fluctuation amplitude captured by the variance, correlation analysis reveals a considerable shift to higher time correlation (long memory) at the late migratory stage. Therefore detrended fluctuation analysis (DFA) was employed to quantify long memory effects and temporal correlation of the impedance time traces given by the scaling exponent *α_DFA_*. In time series analysis DFA is used to evaluate the self-similarity of a signal, here impedance, meaning the pattern looks the same from every scale. A stationary process with slowly decaying correlations is referred to as a stationary process with long memory or long range dependence. For instance, Brownian motion produces a *α_DFA_*-value of 1.5, while larger values indicate longer correlation times associated with long-memory stochastic processes (Peng et al., 1994; Peng et al., 1995). It has been shown that most cells display longer correlation times with *α_DFA_*>1.5 in micromotility assays (Tarantola et al., 2010). In our cases the *α*_DFA_ value amounts up to 1.7 (maximal 2) for migratory PGCs while early PGCs display only a value of about 1.2.

While the variance of impedance noise *σ^2^* denotes the strength of collective motion, the *α_DFA_* value obtained from DFA reflects the emergence of correlations and complex dynamics of cells. It has been found that the spectrum of impedance fluctuations of living cells reveals a power law behavior *f*^−*β*^ with *β*>2.0, which is commonly interpreted as fractional Brownian motion (*β*>1.0) as opposed to fractional Gaussian motion (*β*<1.0) (Cannon et al., 1997). The finding that *β*>1.0 is important for interpretation of *α_DFA_* values and the associated Hurst coefficient *H*, which is related to *α*_DFA_ (*α* = *H*+1 for *β*>1.0) as an indicator for long-memory time series. The Hurst coefficient is frequently used to quantify long-term memory of a signal. While for short-range processes the coupling between successive values decays quickly with distance in an exponential fashion, long-range processes show a power-like decay of the autocorrelation. This is exactly the case in our measurement of PGC motility. The collective shape fluctuations, reorganization of the cytoskeleton and opening/closing of adhesion bonds are the most important factors for higher *α_DFA_* values and therefore Hurst coefficients (Cannon et al., 1997; Peng et al., 1994; Peng et al., 2002; Schneider et al., 2011), implying an increased long-memory effect of the system. This means that the movements are highly predictable in comparison to random Brownian motion.

In summary, both methods reveal similar changes in calculated variances underscoring that the dynamic properties of PGCs change significantly during development. In case of somatic cells, no altered cellular dynamics were detectable, independent of the embryonic stage of development (Fig. 5D).

## Discussion

Germ cells participate in the formation of the gonads, which occurs in a different region than PGC specification (Wylie, 1993). In this context, directional migration of the PGCs to the dorsal crest is required, and it involves the transition from a passive mode of movement during gastrulation with the involuting endoderm, to an active, signal-guided migration. In the present study, we focused on the migratory and adhesive behavior of primordial germ cells during embryonic development of *Xenopus laevis*. Investigation of the physical properties of PGCs during their transition to the motile state could be of more general relevance for the analysis of the mechanisms underlying cell migration. In a new approach based upon the application of biophysical methods, we report on quantitative data for the interaction capacities of PGCs in the early embryonic development of *Xenopus laevis*. During their transition to the migratory stage, PGCs reduce their affinity for fibronectin as well as for the surrounding endodermal cells, correlating with reduced levels of E-cadherin expression in the PGCs. Furthermore, there is a quantifiable increase in cellular dynamics with PGCs gaining motility, and their migratory ability does not depend on the formation of specific interactions with other cells or the extra-cellular matrix, as revealed by *in vitro* migration assays.

We quantified cellular adhesiveness of PGCs by single-cell force spectroscopy (Friedrichs et al., 2010), revealing a stronger attachment to their environment during pre-migratory stages of development in comparison to PGCs isolated from tailbud embryos. This includes a decline in binding affinity to the extracellular matrix protein fibronectin. Comparable results were obtained by Garcia-Castro and coworkers for murine germ cells (García-Castro et al., 1997). These authors made use of an *in vitro* assay and revealed alterations in adhesion strength to the glycoproteins collagen IV, fibronectin and laminin during PGC development (García-Castro et al., 1997). Interestingly, similar to our results, further studies from the same group using a tissue explant system uncovered decreased adhesiveness of mouse PGCs to fibronectin upon reaching the migratory stage (Ffrench-Constant et al., 1991). In contrast to migration within the endoderm, Heasman and coworkers (Heasman et al., 1981) had shown earlier that, in *Xenopus laevis*, fibronectin plays an important role for PGC migration via the dorsal mesentery, reflecting the contribution of the extracellular matrix to PGC guidance. In the experiments described in this communication, only relatively short periods of contact time between cell and substrate were allowed, even though, in the *in vivo* situation, cells are in permanent contact with the surrounding extracellular matrix. However, assembly and disassembly of specific cell-cell contacts is a very dynamic process, especially for migrating cells. Furthermore, an increase of the *in vitro* contact time for a cell with its substrate did not exert much of a change in respect to the quality of the effects observed, but it rather resulted in a significantly higher variability of the strength of adhesion as measured under the conditions of our assay system.

According to the results from our quantitative assay systems, intercellular adhesion can be stronger than the attachment to collagen I or fibronectin, providing strong evidence for the importance of decreasing cell-cell interactions to allow for PGC migration in *X. laevis*. Although several other studies described the importance of cellular adhesion for proper migration of PGCs *in vivo* (Blaser et al., 2005; Goudarzi et al., 2012; Kardash et al., 2010), using under-agarose migration assay, we could show that *Xenopus laevis* primordial germ cells exhibit the principle ability to migrate *in vitro* in the absence of specific interaction complexes forming. Interestingly, in some experiments PGCs isolated form neurula stage embryos could also migrate *in vitro* in the same manner as PGCs isolated from embryos at the tailbud stage. We also observed that the interaction between migrating PGCs and another cell can inhibit migration. Altogether this suggests that cell-cell adhesion is not required for the process of PGC migration itself. In addition, the regulated loss of interactions with neighboring cells via down-regulation of adhesive molecules may be necessary for the initial onset of PGC movement. In the embryo, however, adhesion complex formation might be critical to generate additional traction force for migrating cells. For example, surfaces surrounding migrating cell could not be sufficiently robust and PGCs would thus not be able to generate a critical level of traction force. On the other hand, cell migration might also depend on critical forces to squeeze through the narrow space in between surrounding cells and/or extracellular matrix.

Several studies on zebrafish PGCs demonstrated regulation of intercellular adhesion via E-cadherin as an important element in the context of PGC migration (Blaser et al., 2005; Goudarzi et al., 2012; Kardash et al., 2010). Our qPCR data with *Xenopus* PGCs suggest that the loss of adhesiveness to the surrounding endodermal somatic cells can be explained by developmental down-regulation of specific cell adhesion molecules like E-cadherin in particular. Interestingly, for somatic cells, the level of E-cadherin mRNA remains almost unchanged in the same period of development (Fig. 2). This finding is further supported by single-cell force measurements, revealing no significant changes in the adhesive properties of somatic cells during development (Fig. 1E). As E-cadherin is responsible for the formation of stable intercellular contacts, a strong binding affinity between pre-migratory PGCs and the surrounding somatic cells that declines with the transition to the migratory stage appears to be reasonable. This is indeed also reflected in our single-cell force spectroscopy experiments monitoring a decline in the maximum adhesion force of about 18% after PGCs reach the active migratory stage. Down-regulation of E-cadherin is also known to result in increased migratory behavior of germ cells in *Drosophila melanogaster* (Kunwar et al., 2008). However, activities in addition to those mediated by E-Cadherin, such as via integrins as well as via a cytokine-based adhesion system are known to be involved in the interactions of mouse PGCs with their surrounding somatic cells and adjacent PGCs during migration (reviewed by De Felici, 2000). Additionally, de Felici and coworkers suggest a role for selectin-like molecule mediated adhesion contributing to the interaction between PGCs and somatic cells (De Felici, 2000). Taken together, results obtained by use of very different experimental model systems all support the idea that, due to a lowering of cell adhesion activities, PGC motility is augmented.

Although some of the PGCs from neurula stage embryos could also migrate in an under-agarose migration assay, PGCs isolated from embryos at stages 28–30 generally showed higher ability to migrate under this conditions. Furthermore, using electric cell-substrate impedance sensing (Giaever and Keese, 1984), we depicted an increased impedance noise caused by late PGCs, which we interpret to reflect increased cellular dynamics (Schneider and Janshoff, 2012). As the impedance fluctuations are diminished upon treatment of the cells with cytochalasin B, we attribute the elevated noise in the signal to the increased formation of bleb-like protrusions, which are also known to be the basis for active migration of PGCs in the zebrafish and *Drosophila* (Jaglarz and Howard, 1995). In conjunction with image analysis, we were able to quantify the increase in migratory potential of PGCs during development. The formation of distinct poles of contraction and expansion reflects that the cells are able to push themselves forward, again indicating that cellular adhesion is not the only aspect relevant for the migratory process itself. These data correlate nicely with recently published observations by Terayama and coworkers (Terayama et al., 2013), who demonstrated the change of PGC morphology from spherical with small protrusions at stages 18 and 24 to elongated motile cells at stage 28. Interestingly, the fluctuations of late PGCs are less random than those of early PGCs, displaying long memory according to detrended fluctuation analysis. In terms of biology this means that the cells display more persistent movement in the migratory stage.

In conclusion, we were able to define novel aspects that seem to be relevant for the changes in cellular motility during germ cell development in *Xenopus laevis*. However, a complete description of the gene network that regulates the transition from passive to active PGC migration in *Xenopus* may have many more elements that remain to be discovered.

## Materials and Methods

### Labeling and isolation of *X. laevis* PGCs

*X. laevis* females were primed with 800–1000 U of human chorionic gonadotropin (HCG, Sigma-Aldrich, Munich, Germany) to induce egg-laying. Eggs were *in vitro* fertilized with sliced testis in 0.1× MBSH (5× MBSH: 50 mM HEPES, 440 mM NaCl, 10 mM KCl, 10 mM MgSO_4_, 25 mM NaHCO_3_, 2.05 mM CaCl_2_, 1.65 mM Ca(NO)_3_), dejellied after 1 h with 2% (w/v) cystein hydrochloride (pH 7.9), washed three to five times with 0.1× MBSH and transferred into injection buffer (1–2% (w/v) Ficoll in 1× MBSH). Chimeric *GFP_DELE* mRNA was *in vitro* transcribed from *NotI* (FastDigest, Fermentas, Vilnius, Lithuania) linearized pCS2+gfpDELE plasmid (pCS2+ vector containing EGFP ORF followed by *Xenopus Dead end* (*dnd1*) localization element (DELE)) using mMESSAGE mMACHINE SP6 Kit (Ambion, Austin, Texas, USA). This mRNA was purified by Illustra™ RNA spin MiniRNA Isolation Kit (GE Healthcare, Munich, Germany) according to the manufacturer's protocol. Subsequently, 300–400 pg were injected vegetally into both blastomeres of two-cell stage embryos. At least 1 hour after injections, embryos were transferred into 0.1× MBSH and cultivated at 12.5°C. At developmental stages 17–19 or 28–30 ventral explants containing somatic endodermal cells and primordial germ cells (PGCs) were dissected from the embryos and transferred to 30 mm Petri dishes covered with 1–2% agarose. Explants were dissociated in accutase solution (Sigma-Aldrich, Munich, Germany) for 30 min and washed with 0.8× MBSH. GFP-positive PGCs and GFP-negative somatic endodermal cells were manually sorted using an in-house made eyebrow hair tool.

### Under-agarose migration assay

30 mm Petri dishes were incubated with 2 ml of 5% (w/v) bovine serum albumin (BSA or Albumin Fraction V, Carl Roth GmbH, Karlsruhe, Germany) in 1× PBS overnight, washed three times with 1× PBS and then covered with agarose (0.5% (w/v) in 0.8× MBSH). After the gel polymerized, 0.8× MBSH was added on top to cover the agarose gel. Cells, isolated as described above, were transferred with a pipette under the agarose layer, between the BSA-covered bottom of the Petri dish and the agarose gel. Cells were monitored by time-lapse imaging using a LumarV.12 fluorescence stereomicroscope (Zeiss, Jena, Germany) and the AxioPlan software (Zeiss, Jena, Germany). Tracking was made using Fiji MTrackJ plug-in and analyzed using ImageJ Chemotaxis Tool plug-in.

### Single-cell force spectroscopy

PGCs and somatic endodermal cells were isolated from stage 17–19 or stage 28–30 embryos injected with *GFP_DELE* as described above. All measurements were performed with a Cellhesion200 setup (JPK Instruments, Berlin, Germany) placed on an Olympus IX81 microscope (Olympus, Hamburg, Germany). For cell picking poly-D-lysin coated cantilevers with a nominal spring constant of 0.03 N/m were employed. The spring constant of each cantilever was determined before the measurements using the thermal noise method (Hutter and Bechhoefer, 1993). Before coating the cantilevers with poly-D-lysin, they were washed twice with isopropanol and ultra-pure water and finally cleaned in argon plasma for 1 min. Subsequently, cantilevers were incubated in a 10 µg/ml poly-D-lysin solution (in 1× PBS) for 15 min. To attach either a primordial germ cell or a somatic endodermal cell to the cantilever, a setpoint of 500 pN and a contact time of 30 s before retraction were chosen. To investigate the interaction of either somatic or primordial germ cells with different substrate coatings, Petri dishes were separated into three parts by Liquid-repellent Slide Marker Pen (Super PAP Pen Liquid Blocker, mini; Daito Sangyo Co., Ltd, Osaka, Japan). Each third was coated with either bovine serum albumin (BSA) (5% (w/v) in 1×PBS; Albumin Fraction V, Carl Roth GmbH, Karlsruhe, Germany), fibronectin (50 µg/ml in 1× PBS; F1141-5MG, Sigma-Aldrich, Munich, Germany) or collagen I (50 µg/ml in 0.02 M Acetic acid and 1× PBS; Gibco). Incubation was done overnight, followed by 3× washing with 1× PBS. At least 10 force curves were recorded on each substrate using the same cell on the cantilever. At least three cells were investigated per cell population.

For quantification of cell-cell interactions, we increased the spreading of somatic endodermal cells and primordial germ cells by using fibronectin-coated Petri dishes. Petri dishes were separated into two parts by Liquid-repellent Slide Marker Pen (Super PAP Pen Liquid Blocker, mini; Daito Sangyo Co., Ltd, Osaka, Japan). One part was incubated with fibronectin solution (50 µg/ml in 1× PBS; F1141-5MG, Sigma-Aldrich, Munich, Germany), whereas the second part was saturated by employing BSA (5% (w/v) in 1×PBS; Albumin Fraction V, Carl Roth GmbH, Karlsruhe, Germany) to avoid cell spreading and therefore facilitate picking of the cells. Incubation was done overnight, followed by three times washing with 1× PBS.

Adhesion experiments were carried out with a contact time of 5 sec, an approach/retraction velocity of 3 µm/s and a contact forces of 500 to 1000 pN between the cell attached to the cantilever and the substrate. As a substrate, either a spread cell or one of the different surface coatings mentioned above, was employed.

For a statistical validation of the obtained SCFS data either Wilcoxon rank sum tests or two-tailed t-tests were performed depending on the distribution of the values. The obtained p-values for the SCFS measurements are summarized in supplementary material Tables S1 and S2.

### Electric cell-substrate impedance sensing

Cellular dynamics were assessed using electric cell-substrate impedance sensing (Applied Biophysics, Troy, USA). Therefore, single PGCs or somatic endodermal cells isolated either from stage 17–19 or stage 28–30 embryos injected with *GFP_DELE* were placed onto ultra-small gold electrodes with a diameter of 250 µm. The culture medium (0.8× MBSH) is used as an electrolyte connecting the small electrodes with a huge counter electrode (0.15 cm^2^). The impedance at a frequency of 10 kHz was recorded over time providing information about dynamics of the cell including shape fluctuations. For data analysis the recorded time traces of the impedance |*Z*| were detrended by applying a continuous sliding window of 250 data points and subtracting its arithmetical mean from the central point of the window. This process was included to compensate the effect of thermal drifts. Thereafter, variances of the detrended impedance fluctuations of a 30 min time regime were calculated, which permits monitoring the dynamic behavior of the different cell populations. To inhibit cellular dynamics cytochalasin B (Sigma-Aldrich, Munich, Germany) was added ectopically to a final concentration of 100 µM. Detrended fluctuation analysis (DFA) was carried out according to Peng and coworkers (Peng et al., 1994) using a self-written MatLab script (supplementary material Fig. S6).

### Time-lapse image analysis

Fluorescence microscopy images of migratory and pre-migratory GFP-labeled PGCs were recorded every 15 s for about 15 min. Subsequently, these images are dissected with a self-written picture analysis program based on Matlab (supplementary material Fig. S5). In a first step the original fluorescence pictures are converted into black and white images using individual threshold values for black and white discrimination. Secondly, each picture is subtracted from the prior one to create a difference image. This routine enables us to detect subtle changes in cellular shape over time and allows to distinguish between contracting and expanding areas of the cells. The occurring alterations in area are normalized to the overall cell area at each time point and plotted in percentages. Finally, cellular motility is quantified by calculating the variances of the detected temporal shape fluctuations.

### Quantitative RT-PCR analysis

PGCs and somatic endodermal cells were isolated from stage 17–19 and stage 28–30 embryos as described above. 30 cells from each cell population were used for the preparation of cDNA using SuperScript III CellsDirect cDNA Synthesis System (Invitrogen, Carlsbad, CA, USA) according to the manufacturer's protocol. As a negative control, reverse transcriptase was replaced by the addition of the equal amount of water (RT-) during cDNA preparation from the unsorted group of endodermal cells. This control was included in every cDNA preparation from the corresponding stage. Obtained cDNA was purified using Agencourt AMPure XP paramagnetic beads (Beckman Coulter, Krefeld, Germany) according to the manufacturer's protocol and eluted in 16–50 µL of purification buffer (10 mM Tris-Cl, pH 8.5). Amplification of *Xenopus Primordial germ cell-associated transcript* (*Xpat* or *pgat*) as a PGC-specific gene (Hudson and Woodland, 1998) and β-actin (*actb*) as a positive control were used to monitor quality and contamination of analyzed cell populations using DreamTaq DNA Polymerase (Thermo Fisher Scientific Inc., Schwerte, Germany). Relative size of the amplified products was determined by comparison to the FastRuler Low Range DNA Ladder (Fermentas, Vilnius, Lithuania) Quantitative PCR was done using iQ SYBR Green Supermix (Bio-Rad, Hercules, CA, USA) on CFX96 Real-Time System according to manufacturer's protocol. 1/18 of purified cDNA was used for one PCR reaction. The experiment was done with three independent cDNA preparations. For every cDNA preparation, two technical replicates were performed. RT- from the corresponding stage was used as a template for negative control. Table 1 shows the primers that were employed for PCR (obtained from Sigma-Aldrich, Munich, Germany).

**Table 1.**
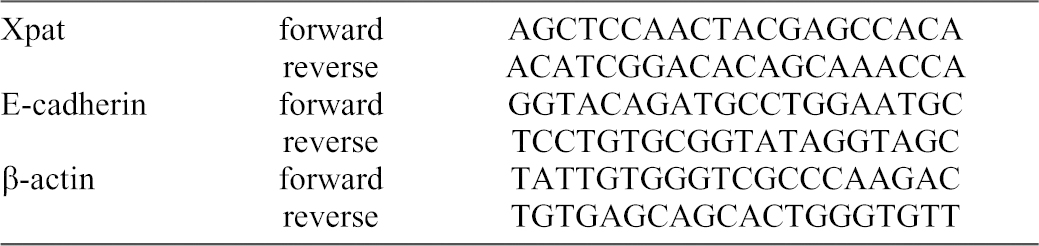
Primers from 5′- to 3′-end used for quantitative PCR.

Analysis of the gene expression was done by ΔCt method with β-actin (*actb*) as a reference gene (Ct_Actb_−Ct_gene_, where Ct is a threshold cycle). Comparison of the expression level between cells isolated from stage 17–19 and stage 28–30 embryos was done by ΔΔCt method (ΔCt_gene,St.28–30_−ΔCt_gene,St.17–19_) (Livak and Schmittgen, 2001).

## Supplementary Material

Supplementary Material
