# Supplementary material for: Migratory and adhesive properties of Xenopus laevis primordial germ cells in vitro
Source: Biol Open. 2013 Nov 6;2(12):1279–87. doi: 10.1242/bio.20135140 (PMC3863412; doi:10.1242/bio.20135140)
Supplement: Supplementary Material [file supp_2_12_1279__index.html]

Migratory and adhesive properties of Xenopus laevis primordial germ cells in vitro — Migratory and adhesive properties of Xenopus laevis primordial germ cells in vitro — Supplementary Material 

# Migratory and adhesive properties of *Xenopus laevis* primordial germ cells *in vitro*

## bio.20135140 Supplementary Material

**Files in this Data Supplement:**

- Supplementary Material - Aliaksandr Dzementsei et al. doi: 10.1242/bio.20135140
