# Supplementary material for: Migratory and adhesive properties of Xenopus laevis primordial germ cells in vitro
Source: Biol Open. 2013 Nov 6;2(12):1279–87. doi: 10.1242/bio.20135140 (PMC3863412; doi:10.1242/bio.20135140)
Supplement: Supplementary Material [file supp_bio.20135140_bio.20135140-s1.pdf]

Biology Open

## Supplementary Material

**Aliaksandr Dzementsei et al. doi: 10.1242/bio.20135140**

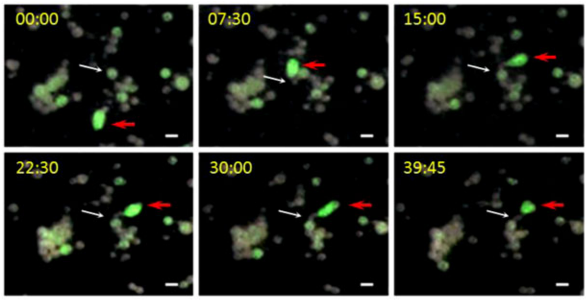


**Fig. S1. Time-lapse images of under-agarose migration assay depicting an anchoring of migrating PGC (red arrow) by another cell (white arrow).** Relative time from the start of the time-lapse is shown in the upper left corner of each image (mm:ss). Scale bar: 20 µm.

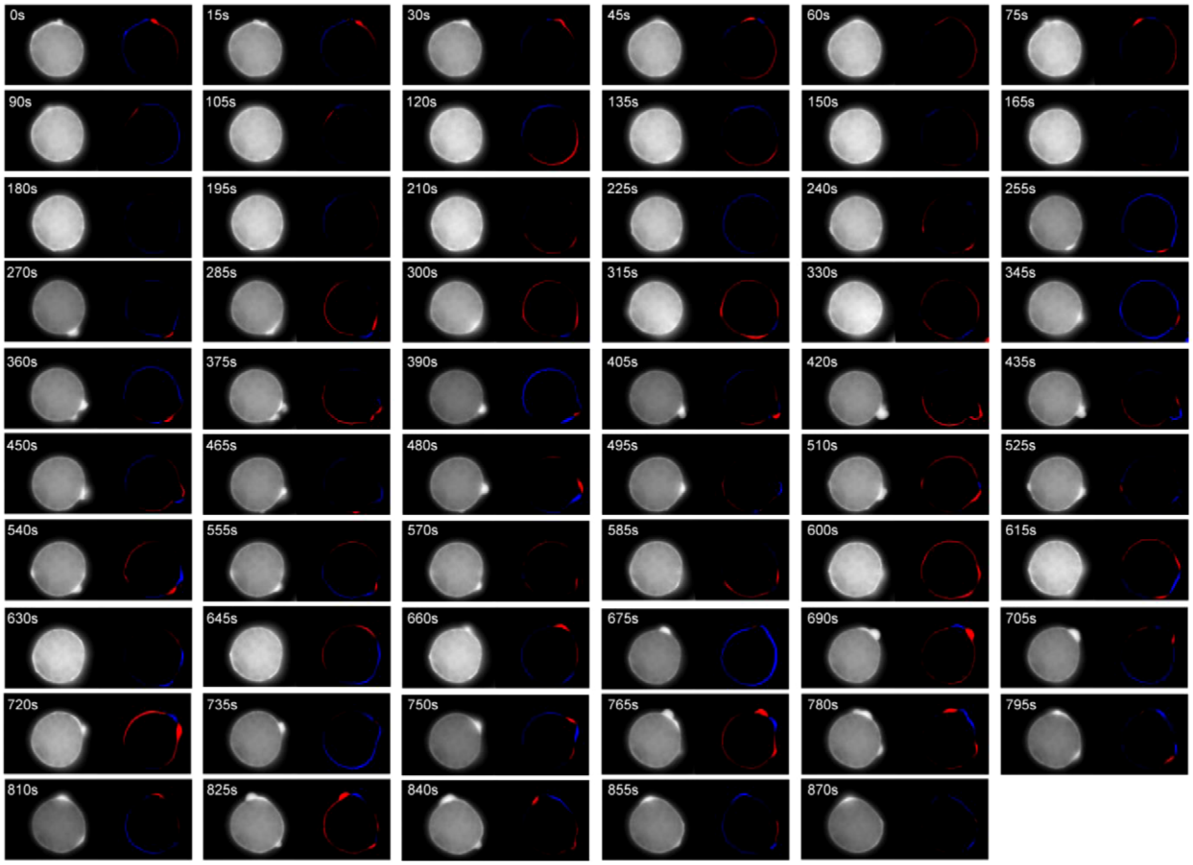


**Fig. S2. Time series of GFP-labeled primordial germ cells within the pre-migratory stage.** Images were taken every 15 s. The shape changes over time were analyzed with a self-written program based on Matlab. Each picture was compared to the prior one revealing regions of expansion - shown in red - and regions of contraction - shown in blue.

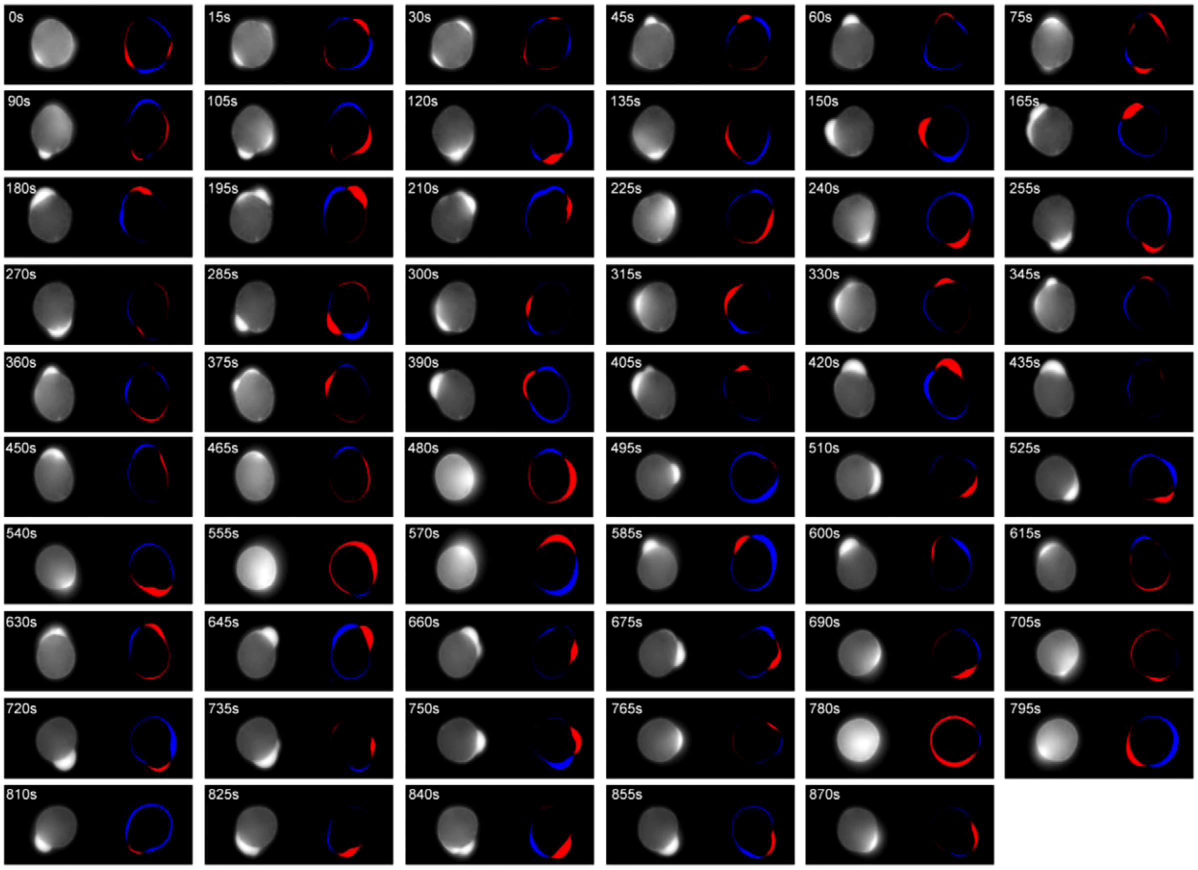


**Fig. S3. Time series of GFP-labeled primordial germ cells within the migratory stage.** Images were taken every 15 s. The shape changes over time were analyzed with a self-written program based on MatLab. Each picture was compared to the prior one revealing regions of expansion - shown in red - and regions of contraction - shown in blue.

**A**

| | Stage 17-19 | | | Stage 28-30 | | |
|---|---|---|---|---|---|---|
| | Total number of PGC | Non-migratory PGC | Migratory PGC | Total number of PGC | Non-migratory PGC | Migratory PGC |
| Experiment 1 | 34 | 22 | 12 | 12 | 5 | 7 |
| Experiment 2 | 110 | 86 | 24 | 201 | 108 | 93 |
| Experiment 3 | 56 | 48 | 8 | 147 | 71 | 76 |
| Experiment 4 | 66 | 47 | 19 | 144 | 63 | 81 |
| Experiment 5 | 77 | 71 | 6 | 64 | 24 | 40 |
| Experiment 6 | 91 | 73 | 18 | | | |

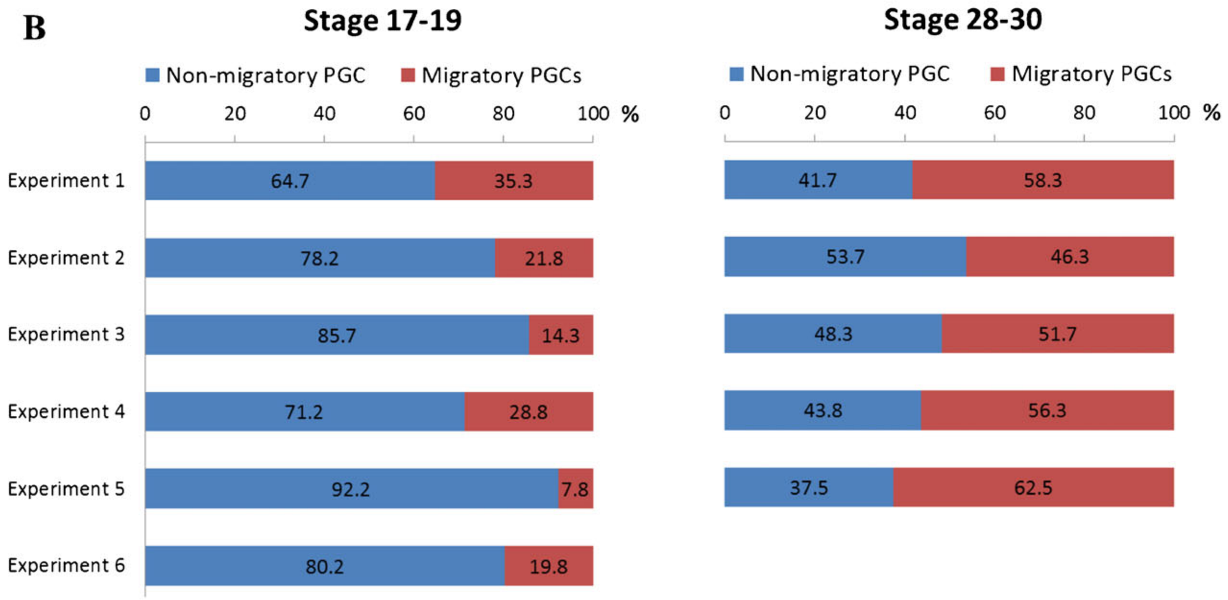


**Fig. S4. Primordial germ cell (PGC) behavior and morphology in the underagarose migration assay.** (A) Total number of PGCs analysed and amount of PGCs assigned for each morphological group. Cells were isolated from stage 17–19 or stage 28–30 embryos. Data are given for each independent experiment. (B) Relative amount of non-migratory and migratory PGCs in each individual experiment.

Biology Open

```
1;
% blebbing.m: Analysis of videos with blebbing cells
%
% requires: imreadBF and imreadBFmeta
%           from LOCI Bioformats Package

% Set parameters: ------------------------------------------

video_filename      = 'PGC_tailbud.zvi';
intensity_threshold = 0.2;
image_fnmask        = 'PGC_tailbud_%02d.png';
result_filename     = 'PGC_tailbud.dat';

color_channel       = 2;      % 1 = red, 2 = green, 3 = blue

% Load image, pre-allocate result arrays: --------------------

info  = imreadBFmeta(video_filename);
video = imreadBF(video_filename, ...    % filename
                [1], ...                % z-planes
                [1:info.nframes], ...   % t-frames
                color_channel);         % colorplane

results  = zeros(info.nframes,1);      % Num. of white pixels
resplus  = zeros(info.nframes,1);      % dto, forward in time
resminus = zeros(info.nframes,1);      % dto, backward in time


% Loop over the frames: ------------------------------------

for frame = 1:info.nframes
    grayscale_img = mat2gray(video(:,:,frame));
    bw_img = grayscale_img > intensity_threshold;
    bw_img = imfill(bw_img,'holes');
    results(frame) = sum(bw_img(:));

    if frame > 1
        dif_img_plus  = bw_img .* ~last_bw_img;
        dif_img_minus = last_bw_img .* ~bw_img;
    else
        dif_img_plus  = bw_img;
        dif_img_minus = bw_img;
    end
    last_bw_img = bw_img;

    resplus(frame)  = sum(dif_img_plus(:))./results(frame);
    resminus(frame) = sum(dif_img_minus(:))./results(frame);

    % Plot current frame and results: ----------------------

    subplot(3,1,1);
    imhist(grayscale_img);
    subplot(3,1,2);



% Combine grayscale, black and white, and difference images

    r = [grayscale_img,bw_img,dif_img_plus];
    g = [grayscale_img,bw_img,0*dif_img_plus];
    b = [grayscale_img,bw_img,dif_img_minus];
    imshow(cat(3,r,g,b));

    if frame > 1
      subplot(3,1,3);
      plot(resplus(2:frame),'r');
      hold on;
      plot(-resminus(2:frame),'b');
      hold off;
      % Combine previous and current grayscale image, and
      % difference image
      r = [last_grayscale_img,grayscale_img,dif_img_plus];
      g = [last_grayscale_img,grayscale_img,0*dif_img_plus];
      b = [last_grayscale_img,grayscale_img,dif_img_minus];
      % Store the result image
      imwrite(cat(3,r,g,b),sprintf(image_fnmask,frame));
    end
    drawnow();
    last_grayscale_img = grayscale_img;
end

% Store the numeric results:
% col 1 : time
% col 2 : pixels changed foreward in time
% col 3 : pixels changed backward in time

dlmwrite(result_filename, ...
         [(2:info.nframes)', resplus(2:end), resminus(2:end)], ...
         ' ');
```

**Fig. S5. Image analysis program for PGCs and somatic cells (Matlab script).**

```
function [Alpha,vari]=DFA_main(data,box,boxplus,boxend)
% data is a time series.
% box is start value, boxplus the increment, and boxend the largest
% boxsize.
% A is the DFA coefficient alpha
% n can be changed to your interest

figure;
n=box:boxplus:boxend;
N1=length(n);
F_n=zeros(N1,1);
 for i=1:N1
     F_n(i)=DFA(data,n(i),1); % DFA function see below
 end

 n=n';
 plot(log(n),log(F_n),'ro');
 xlabel('log(n)');
 ylabel('log(F(n))');
 hold on;
 A=polyfit(log(n(1:end)),log(F_n(1:end)),1);
 xachse=log(n(1:end));
 yachse=A(1)*xachse+A(2);
 plot(xachse,yachse);
 Alpha=A(1);
 return

function output = DFA(data,box,order)
% General function to perform DFA analysis of data set with a specified order. Usually %first oder is
applied

       n=length(data);
       n=floor(n/box);
       n_box = n*box;
       y=zeros(n_box,1);
       Yn=zeros(n_box,1);

       fitcoef=zeros(n,order+1);
       mean1=mean(data(1:n_box));

         for i=1:n_box
                y(i)=sum(data(1:i)-mean1);
         end
       y=y';
         for j=1:n
                fitcoef(j,:)=polyfit(1:box,y(((j-1)*box+1):j*box),order);
                Yn(((j-1)*box+1):j*box)=polyval(fitcoef(j,:),1:box);
         end

       output = sqrt(sum((y'-Yn).^2)/n_box);
```

**Fig. S6. Detrended fluctuation analysis program (Matlab script).**

**Table S1. Calculated p-values of the single-cell force spectroscopy measurements obtained from Fig. 1E.** The interactions of PGCs and somatic cells both from either the early (17–19) or late (28–30) stage of development are compared as indicated. Values are calculated via Wilcoxon rank sum test.

| | PGC-Som (17–19) | Som-Som (28–30) |
|---|---|---|
| PGC-Som (28–30) | $7.6\times10^{-4}$ | $1.5\times10^{-12}$ |
| Som-Som (17–19) | 0.295 | 0.064 |

**Table S2. Calculated p-values of the single-cell force spectroscopy measurements obtained from Fig. 3B.** Either the interaction of either PGCs or somatic cells from the early (17–19) or late (28–30) developmental stage with different substrate coatings or the interaction of the different cell types on the same substrate are compared: (B) BSA, (C) collagen or (F) fibronectin. Values are calculated via students t-test (*) or Wilcoxon rank sum test (**) depending on the distribution of the values.

| | PGCs (17–19) | PGCs (28–30) | Som (17–19) | Som (28–30) |
|---|---|---|---|---|
| BSA-Collagen | 0.045 (*) | 0.17 (**) | 0.005 (**) | 0.406 (*) |
| BSA-Fibronectin | $2.3\times10^{-8}$ (*) | $1.7\times10^{-4}$ (**) | 0.008 (**) | 0.021 (**) |
| Collagen-Fibronectin | $3.2\times10^{-5}$ (*) | 0.037 (**) | $6.9\times10^{-6}$ (**) | 0.004 (**) |
| Late PGCs (B) | $3.1\times10^{-4}$ (**) | / | / | 0.282 (**) |
| Early Som (B) | 0.037 (*) | / | / | 0.050 (*) |
| Late PGCs (C) | $2.9\times10^{-7}$ (**) | / | / | 0.206 (**) |
| Early Som (C) | 0.577 (**) | / | / | $3.3\times10^{-4}$ (**) |
| Late PGCs (F) | 0 (**) | / | / | 0.106 (**) |
| Early Som (F) | 0 (**) | / | / | 0.197 (**) |

Biology Open
